# Decreasing incidence and mortality among hospitalized patients suffering a ventilator-associated pneumonia: Analysis of the Spanish national hospital discharge database from 2010 to 2014

**DOI:** 10.1097/MD.0000000000007625

**Published:** 2017-07-28

**Authors:** Javier de Miguel-Díez, Ana López-de-Andrés, Valentín Hernández-Barrera, Isabel Jiménez-Trujillo, Manuel Méndez-Bailón, José M. de Miguel-Yanes, Benito del Rio-Lopez, Rodrigo Jiménez-García

**Affiliations:** a Respiratory Department, Hospital General Universitario Gregorio Marañón, Facultad de Medicina, Universidad Complutense de Madrid (UCM), Instituto de Investigación Sanitaria Gregorio Marañón (IiSGM); b Preventive Medicine and Public Health Teaching and Research Unit, Health Sciences Faculty, Rey Juan Carlos University; c Internal Medicine Department, Hospital Universitario Clínico San Carlos; d Internal Medicine Department, Hospital General Universitario Gregorio Marañón; e Escuela Técnica Superior de Ingenieros Industriales, Madrid, Spain.

**Keywords:** administrative database, burden of VAP, incidence rate, mortality, ventilator-associated pneumonia

## Abstract

The aim of this study was to describe trends in the incidence and outcomes of ventilator-associated pneumonia (VAP) among hospitalized patients in Spain (2010–2014).

This is a retrospective study using the Spanish national hospital discharge database from year 2010 to 2014. We selected all hospital admissions that had an ICD-9-CM code: 997.31 for VAP in any diagnosis position. We analyzed incidence, sociodemographic and clinical characteristics, procedures, pathogen isolations, and hospital outcomes.

We identified 9336 admissions with patients suffering a VAP. Incidence rates of VAP decreased significantly over time (from 41.7 cases/100,000 inhabitants in 2010 to 40.55 in 2014). The mean Charlson comorbidity index (CCI) was 1.08 ± 0.98 and it did not change significantly during the study period. The most frequent causative agent was *Pseudomonas* and there were not significant differences in the isolation of this microorganism over time. Time trend analyses showed a significant decrease in in-hospital mortality (IHM), from 35.74% in 2010 to 32.81% in 2014. Factor associated with higher IHM included male sex, older age, higher CCI, vein or artery occlusion, pulmonary disease, cancer, undergone surgery, emergency room admission, and readmission.

This study shows that the incidence of VAP among hospitalized patients has decreased in Spain from 2010 to 2014. The IHM has also decreased over the study period. Further investigations are needed to improve the prevention and control of VAP.

## Abstract 2

Supplemental Digital Content is available in the text

## 1 Introduction

Ventilator-associated pneumonia (VAP) is a type of nosocomial pneumonia that occurs in patients who receive more than 48 hours of mechanical ventilation. It is associated with mortality.^[1]^

Over the last years, much improvement has been achieved in understanding the underlying causes and control methods for VAP; however, these infections are still a very frequent health care associated complication.^[2]^

Even if several reports have shown that the incidence of VAP may be decreasing, other studies do not reach this conclusion indicating that the rate is stable over time.^[3–5]^

The evidence shows that VAP results in a significant increment in resource consumption and in patients requiring excess days of hospitalization. Thus, occurrence of VAP increases health system costs.^[6,7]^

Mortality associated with VAP has been reported to range from 33% to 50%. This rate is variable and relies heavily on the underlying medical illness.^[8]^ However, the attributable risk of death has decreased over time, and recently, it has been estimated at 9% to 13%.^[9,10]^ In any case, the incidence, outcomes, and mortality of VAP could vary due to several factors, including the study population, time of onset, etiologic organisms, and adequacy of antibiotic therapy.^[11]^

Despite the significant impact of this disease, studies conducted on the trends of epidemiology of VAP are scarce.^[12–15]^ Unfortunately, the impact of VAP has not been previously determined for the Spanish health care system. Administrative data could be used as primary case-finding methods for this condition.^[16]^ A better understanding of the burden of VAP may help in reducing the incidence and improving patients’ outcomes.

Our objectives were to analyze trends in the incidence, clinical characteristics, and outcomes of VAP in Spain from 2010 to 2014 using the Spanish National Hospital Discharge Database (SNHDD).

## 2 Methods

We conducted an observational retrospective study using the SNHDD.^[17]^ The SNHDD includes information on the sex, age, dates of admission and discharge, up to 14 discharge diagnoses, and up to 20 procedures performed during the hospitalization. The study was conducted with all data included in the SNHDD from January 1, 2010, to December 31, 2014 (5 complete years).

The criteria for diseases and procedures were defined according to the International Classification of Diseases-Ninth Revision, Clinical Modification (ICD-9-CM), which is used in the Spanish SNHDD. We selected admissions for patients with a diagnosis of VAP (ICD-9-CM code: 997.31) in any position.

The Charlson comorbidity index (CCI) was used to assess the clinical characteristics of the patients.^[18]^ We divided patients into 3 categories: low index, which corresponds to patients with no previously recorded disease; medium index, patients with 1 disease category; and high index, patients with 2 or more disease categories.

Irrespectively of the position at the diagnoses coding list, we retrieved data about comorbid specific conditions such as acute myocardial infarction, congestive heart failure, vascular disease, cerebrovascular disease, hemiplegia or paraplegia, dementia, chronic obstructive pulmonary disease (COPD), rheumatoid disease, peptic ulcer, liver disease, renal disease, diabetes, any type of malignancy, metastatic cancer, and acquired immunodeficiency syndrome (AIDS) using the enhanced ICD-9-CM. Furthermore, we identified the following as primary diagnosis: cranial hemorrhage, heart disease, vein or artery occlusion, cranial or spine fracture, pulmonary disease, or nonspecified pneumonia and cancer using the specific ICD-9 codes. We analyzed organ failures, procedures, and pneumonia pathogens documented during hospitalizations for VAP using the same source (see Table 1, Supplemental Content).^[19]^ According to the SNHDD methodology, only those pathogens that are laboratory confirmed can be included in the discharge report.^[17]^ For study purpose, we grouped all species associated with rising IHM in a new variable named “All species that increased IHM.” Hospital outcome variables included emergency room admission, readmission (if the patient had been discharged in the previous 30 days), and the in-hospital mortality (IHM).

### 2.1 Statistical analysis

In order to assess time trends, the incidence rates of hospitalizations with VAP were calculated per 100,000 inhabitants. The denominators were the population, reported on December 31 of each year, according to the Spanish National Institute of Statistics.^[20]^

We estimated the proportion of VAP among mechanically ventilated patients dividing the number of VAP by the total number of hospitalized patient who received invasive mechanical ventilation (ICD MD codes 96.7x in any procedure field) each year.

Variables are described as means with standard deviations or as proportions. Bivariate comparisons were done with Student *t* test, Kruskall–Wallis test, analysis of variance (ANOVA), and χ^2^ test.

Multivariable methods include Poisson regression (incidence) or logistic regression (IHM). The detailed description of the methods can be found elsewhere.^[19]^

The software used for bivariate and multivariable analysis was Stata (Stata, College Station, TX). Statistical significance was set at *P* < .05 (2-tailed).

### 2.2 Ethical aspects

Approval by an ethics committee was not necessary according to the Spanish law. To warranty patient anonymity, the database was provided to us by the Ministry of Health after all patient identifiers were deleted. In accordance with the Spanish legislation, informed consent was not necessary.

## 3 Results

From 2010 to 2014, we identified a total of 9336 admissions with patients suffering a VAP in Spain. Table 1 summarizes the sociodemographic and clinical characteristics of patients included in the study. The mean age was 58.3 ± 18.31 years and there were a predominance of males, without significant changes in age or sex over time. Incidence decreased significantly, from 41.7 cases per 100,000 inhabitants in 2004 to 40.55 in 2014.

**Table 1:**
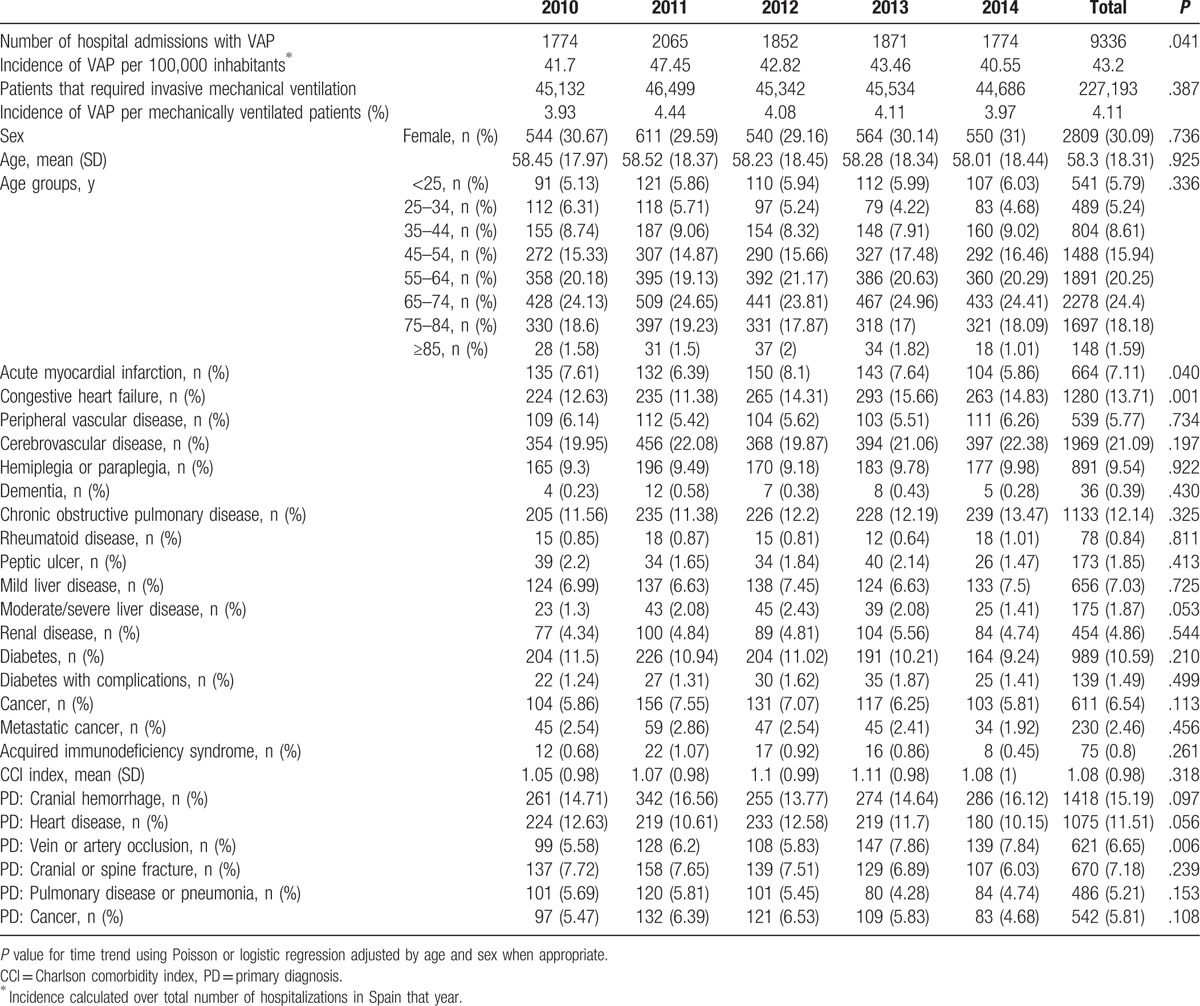
Sociodemographic and clinical characteristics of patients hospitalized who suffered a ventilator-associated pneumonia (VAP) in Spain from 2010 to 2014.

As can be seen in Table 1, the proportion of VAP among mechanically ventilated patients remained stable along the study period with figures around 4%.

The mean CCI index was 1.05 ± 0.98 and it did not change significantly during the study period. The most frequent comorbidities were as follows: cerebrovascular disease (21.09%), congestive heart failure (13.7%), COPD (12.14%), and diabetes (10.59%).

The most common primary diagnosis was cranial hemorrhage (15.19%), followed by heart disease (11.51%), cranial or spine fracture (7.18%), cancer (5.81%), and pulmonary disease or pneumonia (5.21%). We did not find significant variations in the prevalence of these conditions over time.

Procedures, hospital outcomes, acute organ failures, and pathogen isolations of patients hospitalized with VAP are summarized in Table 2. We found an increase in the use of bronchoscopies over time, from 12.74% in 2010 to 16.69% in 2014 (*P* < .05). However, the use of other procedures, such as the thoracentesis or the pleural drainage tube, did not change significantly during the study period.

**Table 2:**
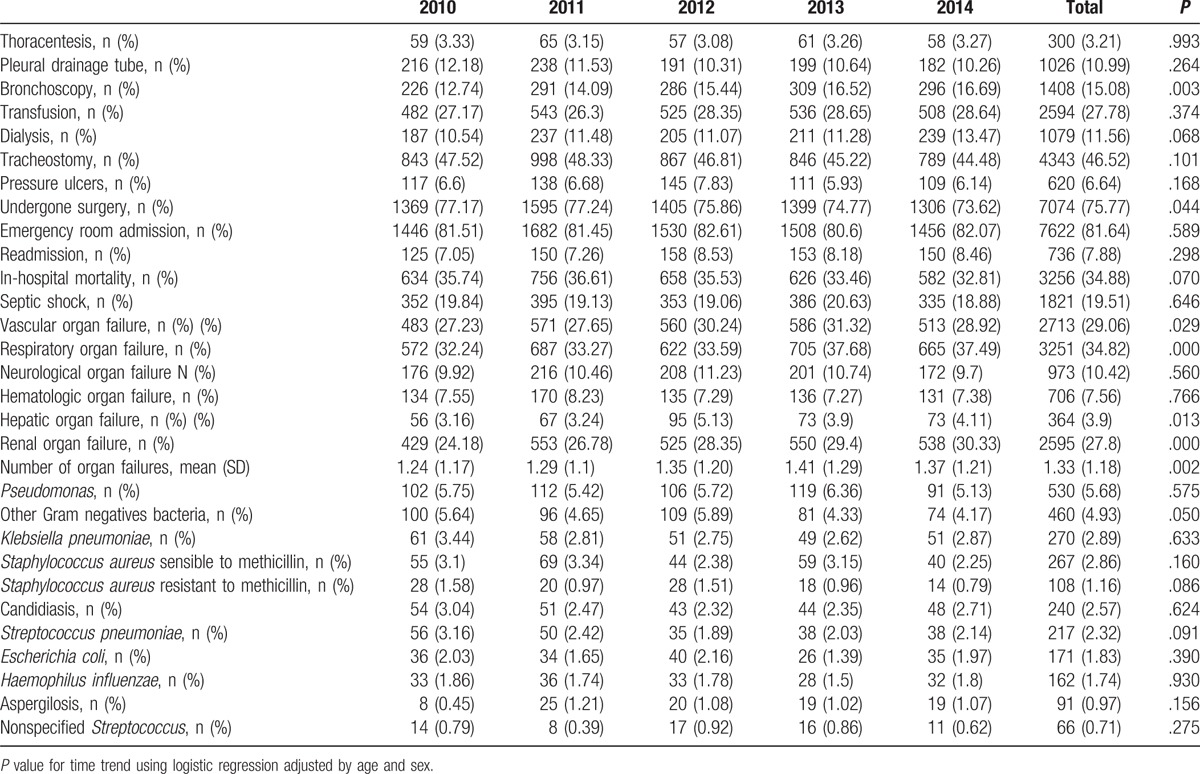
Procedures, hospital outcomes, organ failures, and pathogen isolations of patients hospitalized who suffered a ventilator-associated pneumonia in Spain from 2010 to 2014.

We observed a significant decrease in the percentage of patients who had undergone surgery, from 77.17% in 2010 to 73.62% in 2014. However, we did not detect significant differences in the prevalence of pressure ulcers, septic shock, emergency room admissions, and readmissions (Table 2).

The mean number of acute organ failures significantly increased during the study period, from 1.24 ± 1.17 in 2010 to 1.37 ± 1.21 in 2014. In particular, we observed an increase in the prevalence of cardiovascular failure (from 27.23% in 2010 to 28.92% in 2014), respiratory failure (from 32.24% in 2010 to 37.49% in 2014), renal failure (from 24.18% in 2010 to 30.33% in 2014), and hepatic failure (from 3.16% in 2010 to 4.11% in 2014). However, neurological and hematological failures did not change significantly over time (Table 2).

Within the pathogens analyzed, the most commonly found was *Pseudomonas* (5.68%), followed by other Gram-negative bacteria (4.93%), *Klebsiella pneumonia* (2.89%), *Staphylococcus aureus* susceptible to methicillin (2.86%), candidiasis (2.57%), *Streptococcus pneumoniae* (2.32%), *Escherichia coli* (1.83%), *Haemophilus influenzae* (1.74%), and *S. aureus* resistant to methicillin (1.16%). We did not find significant differences in the isolation of these microorganisms over time. All other pathogens were found in less than 1% of patients (Table 2).

Over the entire time period, IHM was 34.88%. It significantly decreased during the study period, from 35.74% in 2010 to 32.81% in 2014. Table 3 summarizes sociodemographic and clinical characteristics of patients who suffered a VAP according to hospitalization survival. Mortality was higher in males, in older patients, and in those with congestive heart failure, COPD, rheumatoid disease, peptic ulcer, hepatic or renal disease, diabetes with complications, cancer (including metastatic cancer), and AIDS. IHM was also higher when the primary diagnosis was one of the following: vein or artery occlusion, pulmonary disease, and cancer. By contrast, hospital mortality was lower when the primary diagnosis was cranial hemorrhage and cranial or spine fracture.

**Table 3:**
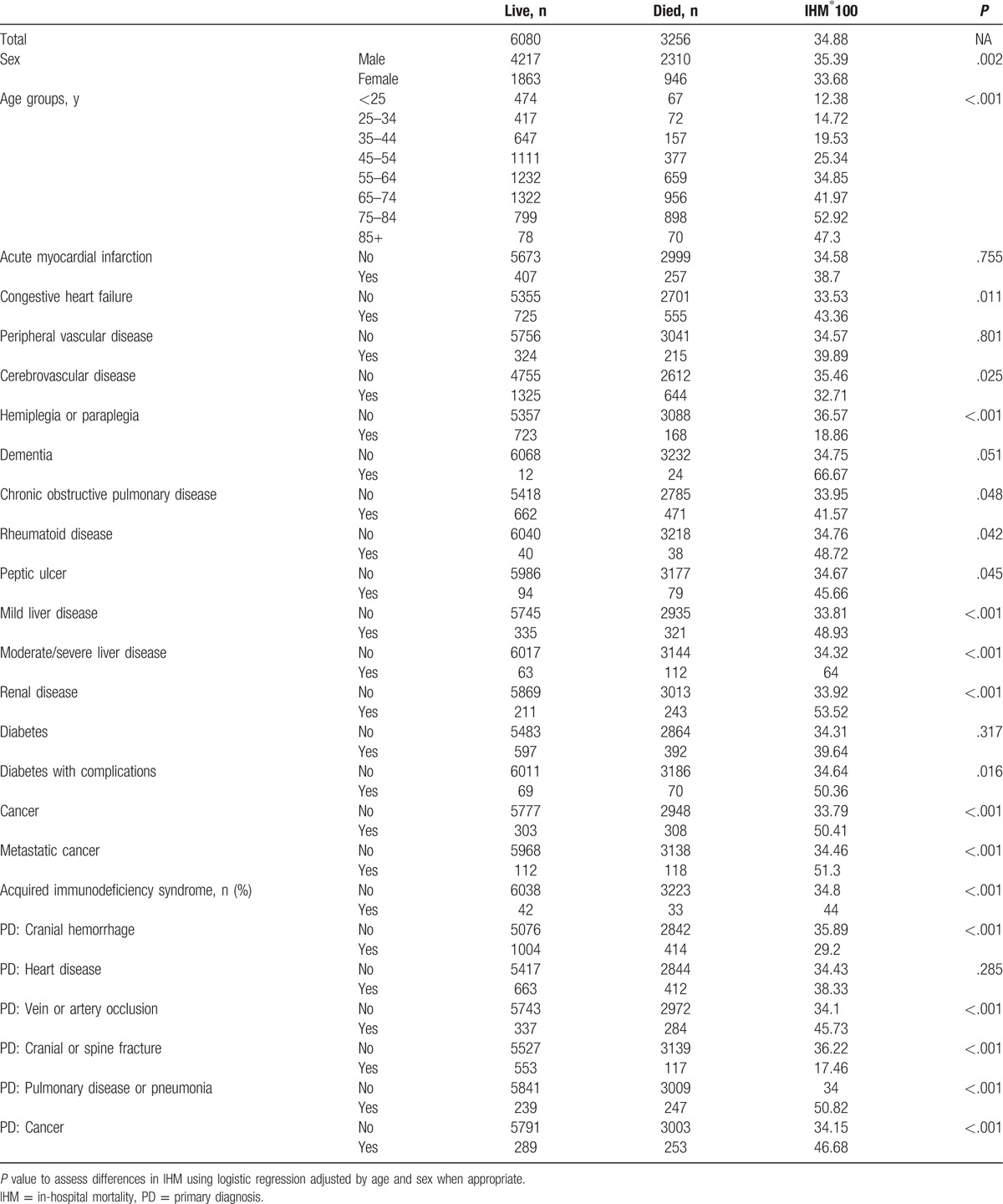
Sociodemographic and clinical characteristics of patient hospitalized who suffered a ventilator-associated pneumonia according to hospitalization survival in Spain, 2010–2014.

Table 4 summarizes procedures, hospital outcomes, acute organ failures, and pathogen isolations in patients hospitalized with VAP according to hospitalization survival. IHM was higher in patients who underwent bronchoscopy, in subjects receiving transfusion, in individuals treated with dialysis, or in those who were readmitted. Patients with acute organ failures (including cardiovascular, respiratory, neurological, hematologic, hepatic, and renal organ failures) also had a higher mortality than those without organ failure. Regarding pathogens isolated, only documented Aspergillosis was associated with increased IHM. As expected, when we grouped all species associated with rising IHM, this variable was associated with higher IHM 39.12% for those with any of these pathogens versus 33.97% for those without.

**Table 4:**
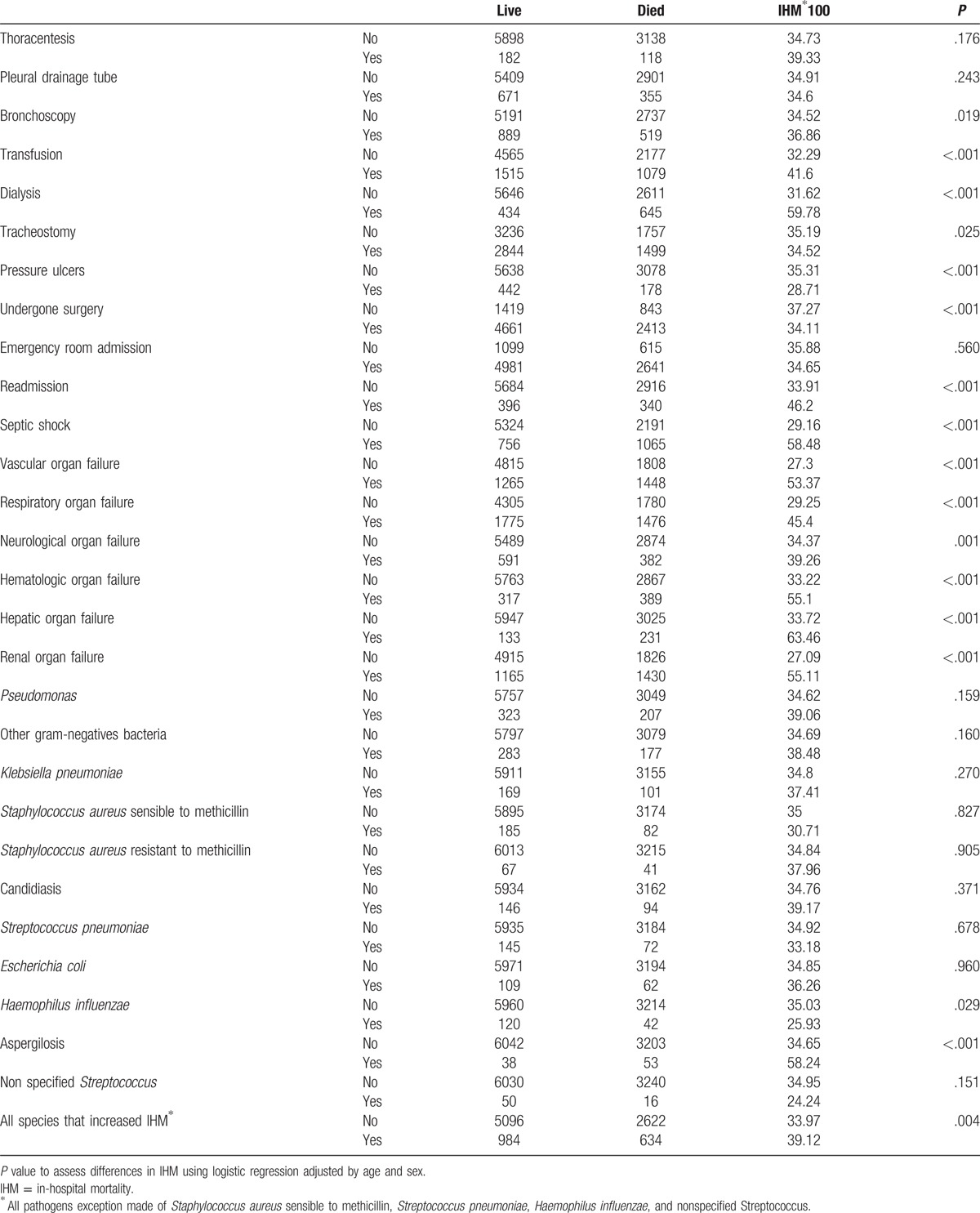
Procedures, hospital outcomes, organ failures, and pathogen isolations in patient hospitalized who suffered a ventilator-associated pneumonia according to hospitalization survival in Spain, 2010–2014.

We can see the results of the multivariate analysis of factors independently associated with IHM among hospitalized patients who suffered VAP in Spain from 2010 to 2014 in Table 5. IHM was significantly higher in males, in older subjects, in patients with comorbidities, in those undergoing surgery, and in patients with one of the following primary diagnosis: vein or artery occlusion, pulmonary disease or pneumonia, and cancer. Mortality was also significantly higher in patients with emergency room admissions and those who were readmitted. By contrast, IHM was significantly lower in patients in which primary diagnosis was cranial hemorrhage and cranial or spine fracture.

**Table 5:**
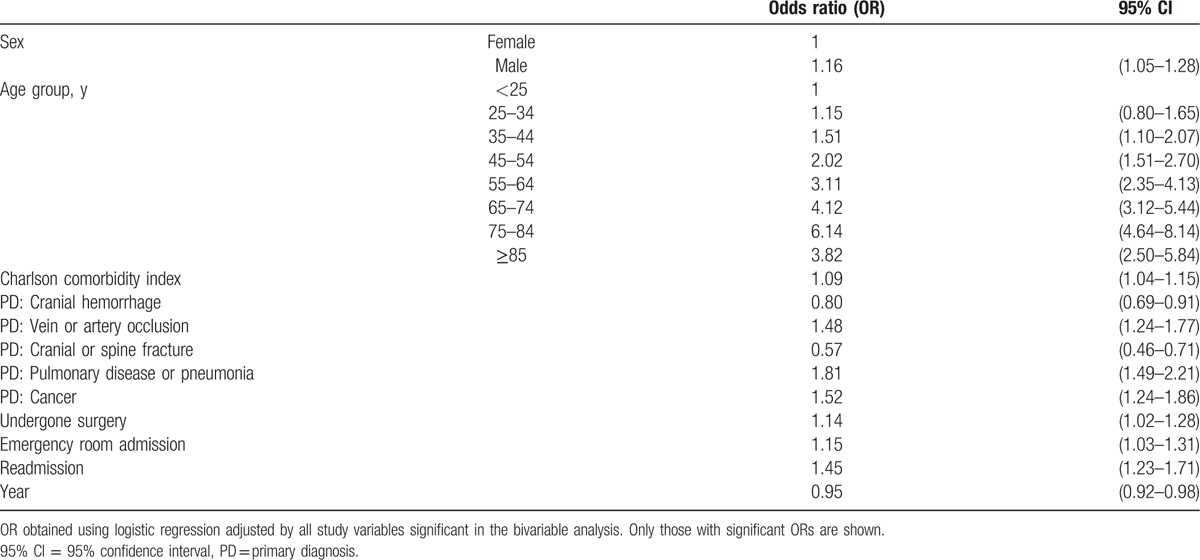
Factors independently associated with in-hospital mortality among patient hospitalized who suffered a ventilator-associated pneumonia in Spain, 2010–2014.

Time-trend analysis showed a significant decrease in IHM in patients admitted with VAP in Spain from 2010 to 2014.

## 4 Discussion

Our results show that the rate of hospitalization for VAP has decreased significantly from 2010 to 2014 in Spain. Other authors have reported similar trends.^[12,21]^ These results might reflect efficiency of preventive measures and critical care practices. In fact, VAP prevention bundle is one of the major strategies used for reducing the incidence of this condition.^[14,22]^ It includes the following components: medical education, use of subglottic suction endotracheal tubes, semi-recumbent position, sedation protocols for rapid weaning, and oral care with chlorhexidine.^[23,24]^ Despite all, it has been demonstrated that there is a wide variability in compliance with VAP-preventive measures across intensive care units in Europe.^[25]^ Apart from compliance with VAP prevention bundles, the rising part of noninvasive ventilation support (noninvasive mechanical ventilation and high flow oxygen therapy) might also explain the decreasing incidence of VAP.

In agreement with other reports, our study showed a male predominance.^[26–28]^ Male sex is one of the nonmodifiable patient-related risk factors for the development of VAP along with others such as preexisting pulmonary disease, AIDS, coma, head trauma, and multiple-organ system failure.^[29]^

The rising use of bronchoscopies in our study is remarkable, whereas the best ways to get microbiological diagnosis in VAP are still discussed. Moreover, it is not clear the why the use of bronchoscopy is associated with mortality. It is possible that this procedure is performed more frequently in patients with a worse clinical course, in order to optimize antibiotic treatment.

We also found an increase in the number of organ failures over time. Specifically, we showed an increased failure of vascular, respiratory, hepatic, and renal organs during the study period. It has been found that multiple organ dysfunction, along with a possible immunosuppression and other underlying diseases, increases the risk of opportunistic infections.^[30]^

According to our results, Gram-negative bacteria were the most frequently isolated pathogens with *Pseudomonas* showing the highest prevalence (5.68%). Furthermore, its dominance did not change over time. Other authors have also found that *Pseudomonas aeruginosa* (PA) is one of the most common bacteria causing VAP,^[31,32]^ with a prevalence of approximately 4%.^[33]^ In addition, VAP caused by PA has been associated with higher case fatality rates than that by other bacteria.^[34]^

The relatively low part of methicillin-resistant *Staphylococcus aureus* has to be pointed out as an element of European epidemiology during the last years that may be different elsewhere.

The low percentage of cases is surprising in which there is an isolated microorganism. It may be because the techniques used to obtain microbiologic specimens, such as bronchoscopy examinations, are not routinely performed in clinical practice, as it has been found in other studies.^[13]^

IHM decreased over time among patients with a diagnosis of VAP in our study, despite the significant increase in mean number of acute organ failures during this period, which could be due to an improvement in the management of these patients over time. These data corroborate to previous studies. In a population-based cohort, the hospital mortality decreased significantly during a 7-year study period.^[13]^ Rosenberger et al ^[35]^ also showed that mortality following an episode of VAP decreased over time and attributed this to advancements in pulmonary and general critical care rather than any specific interventions.

We found a higher mortality in males, in the elderly subgroups, in patients undergoing surgery, and in those with underlying diseases. In fact, CCI was independently associated with an increased risk of IHM in our study. Tseng et al ^[36]^ also showed that high CCI, as well as high Sequential Organ Failure Assessment (SOFA) score, significantly affect hospital mortality in patients with VAP. The isolation of Aspergillus was also associated with increased IHM.

In our study of VAP patients, IHM was higher when principal diagnosis was occlusion of a vessel, pulmonary disease, or cancer. Malignancy has also been reported as a prognostic indicator of hospital mortality in a recent study.^[37]^ Patients with cancer are at a high risk of infections and subsequent complications. Identified risk factors for VAP in cancer patients include age (≥65 years), surgery, and tracheostomy.^[38]^ On the contrary, IHM was lower when principal diagnosis was cranial hemorrhage or cranial or spinal fracture. Cinotti et al ^[39]^ found that among patients suffering from subarachnoid hemorrhage, longer ICU stay and time with mechanical ventilation increased the risk of VAP, but not of mortality.

Other factor independently associated with IHM mortality among patients hospitalized with VAP in the present study was emergency room admission. Prior reports that have examined the effect of boarding on intensive care unit outcomes, including VAP, have found an association between increased emergency department LOHS with poor outcome.^[40,41]^ Thus, for example, it has been demonstrated that in blunt trauma patients who are emergently intubated, increased emergency department length of stay is an independent risk factor for pneumonia. VAP interventions, successful in the intensive care unit, should be implemented early in the hospital course, and efforts should be made to minimize hospital crowding and emergency department length of stay.^[40]^

Readmission increases the risk of IHM after VAP in our population. In a retrospective case–control study, patients in the VAP group had a greater number of readmissions than the control group patients.^[42]^

This study examines the impact of VAP across the Spanish health care system rather than at an institutional level. The strengths of our findings lie in the large sample size, the 5-year follow-up period, and the standardized methodology, which has been used to investigate VAP and its complications.^[7,42]^ Nevertheless, our study has some limitations. Our data source was the SNHDD, an administrative database that uses information the physician has included in the discharge report. Administrative data can be inaccurate for detection of hospital-acquired infections, including VAP. A systematic review by Goto et al ^[16]^ included 2 studies for VAP and both reported low to moderate sensitivity (42–72%) and moderate to high specificity (82–92%). It is unclear how this may affect the results of the study. Regardless, as the methodology has remained constant throughout the study, we consider that changes detected over time are valuable. Furthermore, as the database does not include dates for diagnosis or procedures, it fails to establish a temporal relationship between the procedures, surgeries, septic shock, and pressure ulcers in the patients with VAP. Also, it is not possible to determine whether comorbidities were already present when the patient was admitted or may have appeared during the hospital stay. However, it is logical to think that chronic conditions (i.e., diabetes, COPD, etc) were present by the time of admission. Finally, our findings are limited by the lack of data, including, among others, severity of illness, antimicrobial therapy, specimen quality, and length of stay in the intensive care unit. These and other factors that may influence in VAP outcomes could not be analyzed.

In addition, we cannot identify whether changes in the use of strategies to prevent VAP during the study period may have had an influence on the results.

The study is limited by the fact that if a patient was admitted in the same year twice or more times, this event could not be detected; this is a consequence of the anonymity of the database. Furthermore, if a patient is transferred from one to another hospital, it would also be counted as 2 different admissions. However, the SNHDD has proved to be useful for epidemiological investigation, covers over 98% of hospital admission in Spain, and the Ministry of Health conducts periodical audits to warrantee its validity.^[17,43]^ The code for VAP was first used in the SNHDD in 2010, so some underreporting in the first years could be expected.

In conclusion, this study shows that the incidence of VAP among hospitalized patients has decreased in Spain from 2010 to 2014. The IHM has also decreased over the study period. These results indicate that both prevention and management of VAP have probably got better in Spain during the study period.

## Supplementary Material

Supplemental Digital Content
