# Supplementary material for: Decreasing incidence and mortality among hospitalized patients suffering a ventilator-associated pneumonia: Analysis of the Spanish national hospital discharge database from 2010 to 2014
Source: Medicine (Baltimore). 2017 Jul 28;96(30):e7625. doi: 10.1097/MD.0000000000007625 (PMC5627849; doi:10.1097/MD.0000000000007625)
Supplement: Supplemental Digital Content [file medi-96-e7625-s001.docx]

**Supplementary Table 1.** Main codes used to identify diseases, procedures and pathogen isolations according to the International Classification of Diseases-Ninth Revision, Clinical Modification (ICD-9-CM).

|  | **ICD-9-CM Code** |
| --- | --- |
|  |  |
| Primary diagnosis | Cranial hemorrhage (430, 431, 852), heart disease (410, 414, 424, 427, 428), vein or artery occlusion (038, 434), cranial or spine fracture (800, 801, 806), pulmonary disease or pneumonia unespecified (486, 518), cancer (140.x-172.x, 174.x-195.8, 200.x-208.x, 238.6). |
| Organ failures | Septic shock (785.52), cardiovascular failure (458, 458.0, 458.1, 458.2, 458.21, 458.29, 458.8, 458.9, 785.5, 785.50, 785.51, 785.52, 785.59), respiratory failure (96.7, 189.1, 518.81, 518.82, 786.09), neurological failure (293.0, 293.9, 348.1, 348.3, 780.01), hematologic failure (286.6, 286.9, 287.3, 287.4, 287.5), hepatic failure (570, 572.2, 573.4), renal failure (584.5, 584.6, 584.7, 584.8, 584.9). |
| Diagnostic or therapeutic procedures | Thoracentesis (34.91), pleural drainage tube (34.0, 34.01, 34.02, 34.03, 34.04, 34.05, 34.06, 34.07, 34.08, 34.09), bronchoscopy (33.21, 33.22, 33.23, 33.24), transfusion (99.00, 99.01, 99.02, 99.03, 99.04, 99.05, 99.06, 99.07, 99.080), dialysis (39.95, 54.98), tracheostomy (31.1), pressure ulcers (707.xx). |
| Pathogen isolations | *Pseudomonas* (482.1), other Gram negative bacteria (482.83), *Klebsiella pneumoniae* (482.0), *Staphylococcus aureus* sensible to methicillin (482.41), *Staphylococcus aureus* resistent to methicillin (482.42), candidiasis (112.4), *Streptococcus pneumoniae* (481), *Escherichia coli* (482.82), *Haemophilus influenzae* (482.2), *Aspergillus* (117.3), non specificied *Streptococcus* (482.3). |

Codes used for the CCI are those reported by Deyo et al in “Deyo RA, Cherkin DC, Ciol MA. Adapting a clinical comorbidity index for use with ICD-9-CM administrative databases. J Clin Epidemiol. 1992;45(6):613-9.”
